# Single-Cell Transcriptome Analysis of CD34^+^ Stem Cell-Derived Myeloid Cells Infected With Human Cytomegalovirus

**DOI:** 10.3389/fmicb.2019.00577

**Published:** 2019-03-21

**Authors:** Melissa Galinato, Kristen Shimoda, Alexis Aguiar, Fiona Hennig, Dario Boffelli, Michael A. McVoy, Laura Hertel

**Affiliations:** ^1^Center for Immunobiology and Vaccine Development, Children’s Hospital Oakland Research Institute, Oakland, CA, United States; ^2^Center for Genetics, Children’s Hospital Oakland Research Institute, Oakland, CA, United States; ^3^Department of Pediatrics, Virginia Commonwealth University, Richmond, VA, United States

**Keywords:** single-cell, tropism, cytomegalovirus, myeloid, 10× genomics, RNAseq

## Abstract

Myeloid cells are important sites of lytic and latent infection by human cytomegalovirus (CMV). We previously showed that only a small subset of myeloid cells differentiated from CD34^+^ hematopoietic stem cells is permissive to CMV replication, underscoring the heterogeneous nature of these populations. The exact identity of resistant and permissive cell types, and the cellular features characterizing the latter, however, could not be dissected using averaging transcriptional analysis tools such as microarrays and, hence, remained enigmatic. Here, we profile the transcriptomes of ∼7000 individual cells at day 1 post-infection using the 10× genomics platform. We show that viral transcripts are detectable in the majority of the cells, suggesting that virion entry is unlikely to be the main target of cellular restriction mechanisms. We further show that viral replication occurs in a small but specific sub-group of cells transcriptionally related to, and likely derived from, a cluster of cells expressing markers of Colony Forming Unit – Granulocyte, Erythrocyte, Monocyte, Megakaryocyte (CFU-GEMM) oligopotent progenitors. Compared to the remainder of the population, CFU-GEMM cells are enriched in transcripts with functions in mitochondrial energy production, cell proliferation, RNA processing and protein synthesis, and express similar or higher levels of interferon-related genes. While expression levels of the former are maintained in infected cells, the latter are strongly down-regulated. We thus propose that the preferential infection of CFU-GEMM cells may be due to the presence of a pre-established pro-viral environment, requiring minimal optimization efforts from viral effectors, rather than to the absence of specific restriction factors. Together, these findings identify a potentially new population of myeloid cells permissive to CMV replication, and provide a possible rationale for their preferential infection.

## Introduction

Infection by human cytomegalovirus (CMV) is common and usually asymptomatic in healthy individuals, but can be the source of serious disease in hosts with naïve or compromised immune functions such as fetuses, newborns, AIDS patients, and solid organ or bone marrow transplant recipients (Pass, 2001; Griffiths et al., 2013). CD34^+^ hematopoietic stem cells (HSC) and derived monocytes, macrophages, and dendritic cells are important sites of CMV latency and reactivation, as well as of lytic infection *in vivo* (Hertel, 2014; Sinclair and Reeves, 2014; Stevenson et al., 2014; Dupont and Reeves, 2016). CMV interactions with these cells have thus been intensively studied, using a variety of different cell culture models (Ibanez et al., 1991; Kondo et al., 1994; Goodrum et al., 2002; Hertel et al., 2003; Reeves et al., 2005).

We previously showed that culture of cord blood CD34^+^ HSC in the presence of cytokines known to instruct their differentiation into Langerhans cells (Strobl et al., 1997, 2018), gives rise to a population of cells capable of restricting infection progress at multiple steps of the viral replication cycle (Lauron et al., 2014; Coronel et al., 2015, 2016), thus providing an outstanding model to study the cellular determinants of CMV tropism. Their intrinsic heterogeneity, however, has thus far precluded the identification of cellular factors supporting or restricting infection using averaging gene expression analysis tools such as microarrays. Here, we took advantage of the most recent developments in single-cell RNA sequencing technologies to provide the first transcriptional profiling of a population of myeloid cells permissive to CMV lytic infection, and the first comparison of cellular gene expression changes occurring in cells expressing high levels of a large variety of viral genes versus cells containing lower levels (viral transcript low) or undetectable levels (viral transcript^-^) of viral transcripts, all co-existing in the same population.

We show that: (1) more than half of the cells contain detectable viral transcripts at day 1 post-infection, with only a small minority (∼2%) displaying an expression pattern consistent with progression to lytic replication. This indicates that restrictions to viral entry may contribute to, but are not the main determinants of resistance; (2) lytically infected cells are transcriptionally related to a specific cluster of cells with the hallmarks of Colony Forming Unit – Granulocyte, Erythrocyte, Monocyte, Megakaryocyte (CFU-GEMM) oligopotent progenitors, suggesting that this type of cells may be a previously unidentified target of CMV lytic infection; (3) compared to the remainder of the population, CFU-GEMM cells express similar or higher levels of interferon (IFN)-related genes with anti-viral roles, which are strongly down-regulated in infected cells, indicating that CFU-GEMM cells are not defective in their ability to recognize and respond to CMV infection; (4) also compared to the remainder of the population, CFU-GEMM cells are enriched in transcripts encoding proteins involved in mitochondrial energy production, S-phase control, and RNA and protein production. Expression levels of these genes remain largely unchanged in infected cells, suggesting that preferential infection of CFU-GEMM progenitors is likely due to the presence of a transcriptional landscape already optimized for viral replication, and requiring little conditioning effort from viral effectors, rather than to an intrinsic inability to recognize and respond to the presence of viral products.

## Materials and Methods

### Cells and Virus

Umbilical cord blood CD34^+^ HSC were purchased from STEMCELL Technologies Inc., Vancouver, Canada and pre-amplified in α-Minimum Essential Medium (Thermo Fisher Scientific, Waltham, MA, United States) supplemented with 20% heat-inactivated fetal bovine serum (FBS, Gibco, Fisher Scientific, Waltham, MA, United States), 375 ng/ml of Flt3 ligand (FL), 50 ng/ml of stem cell factor (SCF) and 50 ng/ml of thrombopoietin (TPO) for 8–10 days at a density of 1 × 10^4^ cells/well in 48-well tissue culture plates. Cells were then differentiated in serum-free X-VIVO 15 medium (Lonza/BioWhittaker, Allendale, NJ, United States) supplemented with 1,500 IU/ml of granulocyte-macrophage colony-stimulating factor (GM-CSF, Leukine Sargramostim), 150 ng/ml of FL, 10 ng/ml of SCF, 2.5 ng/ml of tumor necrosis factor-α (TNF-α), and 0.5 ng/ml of transforming growth factor β1 for 8 days at a density of 1 × 10^5^ cells/well in 48-well plates. Activation of differentiated cells was then induced by exposure to X-VIVO 15 medium containing 10% standard FBS (US origin, Gibco, Fisher Scientific, Waltham, MA, United States), 1,500 IU/ml of GM-CSF, 200 ng/ml of CD40 ligand (CD40L) and 500 ng/ml of lipopolysaccharide (LPS, Sigma-Aldrich, St. Louis, MO, United States) for 2 days at a density of 1 × 10^5^ cells/well in 48-well plates. All cytokines were from Peprotech, Rocky Hill, NJ, United States.

Human foreskin fibroblasts, a kind gift from Dr. E. S. Mocarski, were propagated in Dulbecco’s Modified Eagle Medium (Corning Cellgro, UCSF CCF, San Francisco, CA, United States) supplemented with 10% fetal clone serum III, 100 U/ml penicillin, 100 μg/ml streptomycin, 4 mM HEPES (all from HyClone, Fisher Scientific, Pittsburgh, PA), and 1 mM sodium pyruvate (Corning Cellgro, UCSF CCF, San Francisco, CA, United States). CMV strain TB40/E, a gift from C. Sinzger (University of Ulm, Ulm, Germany), was propagated on fibroblasts and purified by ultracentrifugation as previously described (Hertel and Mocarski, 2004).

### Myeloid Cell Infection

Differentiated myeloid cell populations were exposed to TB40/E at a calculated multiplicity of infection (MOI) of 10 pfu/cell for 4 h, washed three times and further cultured for 10 days. Cells were harvested on days 2, 4, 6, 8, and 10 post-infection (pi), counted, and used in immunofluorescence staining analyses and titration assays.

### Immunofluorescence Staining Analyses

Cell staining was performed as previously described (Coronel et al., 2016). Briefly, cytospin preparations were fixed in 1.5% formaldehyde for 30 min, permeabilized in 0.5% Triton-X 100 for 20 min, and blocked in 40% FBS/40% goat serum for 30 min before incubation with antibodies directed against the viral immediate-early proteins 1 and 2 (IE1/IE2, MAb810, 1:600, or AF488 MAB810X, 1:200, Millipore, Temecula, CA, United States), UL84 (1:500, Virusys, Taneytown, MD), UL44 (1:200, Virusys, Taneytown, MD), or UL57 (1:100, Virusys, Taneytown, MD, United States) for 1 h, followed by secondary antibodies conjugated to Alexa-Fluor 488 or Alexa-Fluor 594 (1:200, Invitrogen, Carlsbad, CA, United States, and Jackson Immunoresearch, West Grove, PA, United States) for another hour. Nuclei were labeled with Hoechst 33342 (0.2 mg/ml; Molecular Probes, Eugene, OR, United States) for three min. Samples were viewed using a Nikon Eclipse E600 fluorescence microscope equipped with Ocular imaging software.

### Virus Titrations

Cell-associated virus was released from pelleted myeloid cells by sonication for ∼5–10 s on ice using a Branson Ultrasonics Sonifier 150 and incubated with fibroblasts for 1 h. Infected fibroblasts were stained for IE1/IE2 expression at 24 h post-infection.

### Statistical Analysis

All data were analyzed using Prism 7 (GraphPad Software). Unpaired *t*-tests were used to compare data from non-activated and activated cells in Figure 3. Differences were considered significant at *P* < 0.05. The Wilcoxon signed rank sum test was used to compare median ratio values from data distributions with a hypothetical median of zero.

### Single-Cell RNA-Seq Generation and Analysis

Activated myeloid cells differentiated from the CD34^+^ HSC of a representative donor (113G) were infected with TB40/E at an MOI of 10 pfu/cell, washed five times, and further incubated for 24 h. Cells were then processed through the Chromium Single-cell 3′ v2 Library Kit (10× Genomics) by the Genetic Resources Core Facility Cell Center and BioRepository, Johns Hopkins University, Baltimore, MD, United States. Briefly, 10,000 cells were loaded onto a single channel of the 10× Chromium Controller. Messenger RNA from approximately ∼7,000 cells, captured and lysed within nanoliter-sized gel beads in emulsion, was reverse transcribed and barcoded using polyA primers with unique molecular identifier sequences before being pooled, amplified, and used for library preparation. The library was then sequenced in two lanes of an Illumina HiSeq 2500 Rapid Flowcell system. A summary of all sequencing parameters and outcomes is provided in Supplementary Figure S1. Demultiplexing of the bcl file into a FASTQ file was performed using Cell Ranger v1.2 mkfastq software, and alignments to human (hg19) or TB40E (NCBI EF999921.1) genome reference sequences were performed using STAR (Dobin et al., 2013). Dimensionality reduction of data was performed by principal component analysis using *N* = 10 principal components, and reduced data were visualized in two dimensions using the *t*-SNE non-linear dimensionality reduction method (van der Maaten and Hinton, 2008). Clustering for expression similarity was performed using both graph-based and *K*-means (with *K* = 10 clusters) methods by Cell Ranger (CellRanger, 2018). Clusters and differential expression analyses generated by Cell Ranger were then visualized using Loupe^TM^ Cell Browser (CellBrowser, 2018). For each gene in each cluster, three values were computed and reported in supplemental datasets: (1) the mean number of unique molecular identifier counts; (2) the log2 fold-change of each gene’s expression in cluster x relative to other clusters and (3) the *p*-value denoting significance of each gene’s expression in cluster × relative to other clusters, adjusted to account for the number of hypotheses (i.e., genes) being tested.

### Monocle Clustering and Single Cell Ordering in Pseudotime

Cells belonging to the cluster 7, erythro, mono, MDDC, CMV^+^, promyelo, act neut, and sub-cluster 3 groups depicted in Figure 3C were used for pseudotime analysis. Gene-cell matrices produced by Cell Ranger were loaded into R with cellrangerRkit^1^ and pseudo-temporal assignment was performed with Monocle version 2.99.0 (Trapnell et al., 2014) using *N* = 5 principal components. Marker genes were found using Seurat’s FindAllMarkers function (Butler et al., 2018), and groups were identified based on the expression of gene markers from Figure 3C and Supplementary Figure S4. The root of the tree was manually selected using orderCells from Monocle, defined by the point of origin of the majority of the branches.

### Data Availability

All single-cell data files are deposited in Gene Expression Omnibus under accession number GSE124334.

## Results

### CD34^+^ HSC-Derived Myeloid Cell Populations Restrict CMV Infection at Multiple Steps of the Viral Replication Cycle

To identify cellular factors potentially involved in regulating myeloid cells permissiveness to CMV infection, we sought to analyze the transcriptome of a representative population of activated cells differentiated from CD34^+^ HSC *in vitro*. We specifically focused on activated cells because of their ability to restrict progression of viral replication after infection onset (Coronel et al., 2016).

**FIGURE 1:**
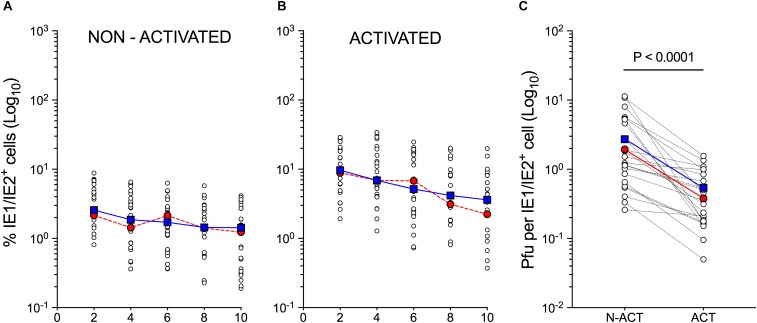
Susceptibility to CMV infection of non-activated and activated myeloid cell populations differentiated from cord blood CD34^+^ HSC. Non-activated and activated myeloid cell populations differentiated from the CD34^+^ HSC of twelve different cord blood donors were exposed to CMV strain TB40/E at an MOI of 10 and analyzed at days 2, 4, 6, 8, and 10 pi. **(A,B)** Percentage of IE1/IE2^+^ cells (Log_10_) present in each population as determined by immunofluorescence staining analyses. **(C)** Peak (days 6–10) intracellular progeny yields as quantified by titration assays of cell sonicates from paired cultures of non-activated (N-ACT) and activated (ACT) cell populations. Open circles represent data from individual donors obtained in separate experiments. Some donors were used more than once. Blue squares indicate median values at each time point. Red circles depict data obtained from the CD34^+^ HSC of representative donor 113G.

To select a representative population, the CD34^+^ HSC isolated from the cord blood of twelve different donors were separately cultured in the presence of cytokines known to promote the development of Langerhans cells (Strobl et al., 1997; Strobl et al., 2018). Differentiated cells were then activated by exposure to GM-CSF, FBS, CD40L and LPS, and infected with the endo/epitheliotropic strain TB40/E at an MOI of 10 pfu/cell. Consistent with our previously published data (Lauron et al., 2014; Coronel et al., 2015, 2016), cell numbers did not increase over time (not shown), and only 3 ± 1.5% of non-activated, but 10 ± 5% of activated cells expressed the viral IE1/IE2 proteins at day 2 pi (Figure 1A,B). Despite containing higher numbers of IE1/IE2^+^ cells at each time point, activated populations produced lower progeny amounts per IE1/IE2^+^ cell, with consistently different mean peak yields (*P* < 0.0001, Wilcoxon matched-pair signed rank test, Figure 1C).

As data derived from activated cells differentiated from the CD34^+^ HSC of donor 113G (Figure 1, red circles) closely mirrored the median values obtained from all tested populations (Figure 1, blue squares), this population was selected as representative, and subjected to single-cell RNA sequencing at day 1 using the 10× Genomics Chromium platform. Activated cells were chosen to ensure data collection from sufficient numbers of infected cells and to facilitate the identification of potential cellular mediators of viral tropism, whereas the day 1 time point was selected to allow sufficient time for viral transcripts to accumulate to detectable levels, while limiting the extent of virus-induced changes to the cellular transcriptome, in order to enable the identification of specific cell types within the infected population.

A median of 2,305 genes and 10,627 transcripts were detected in the 6,837 cells profiled, and the total number of genes with at least one count in any cell was 20,899. After reduction by principal components analysis, data was visualized in two dimensions using the *t*-distributed stochastic neighbor embedding (*t*-SNE) algorithm (van der Maaten and Hinton, 2008), which displays cells with similar transcriptional profiles as nearby points, and cells with dissimilar transcriptional profiles as distant points with high probability. Cells thus represented on *t*-SNE plots were then interrogated for their content in specific gene transcripts using Loupe^TM^ Cell Browser.

### Viral Transcripts Are Detected in the Majority of the Cells, but Their Presence Is Not Associated With Expression of Specific Cellular Genes

**FIGURE 2:**
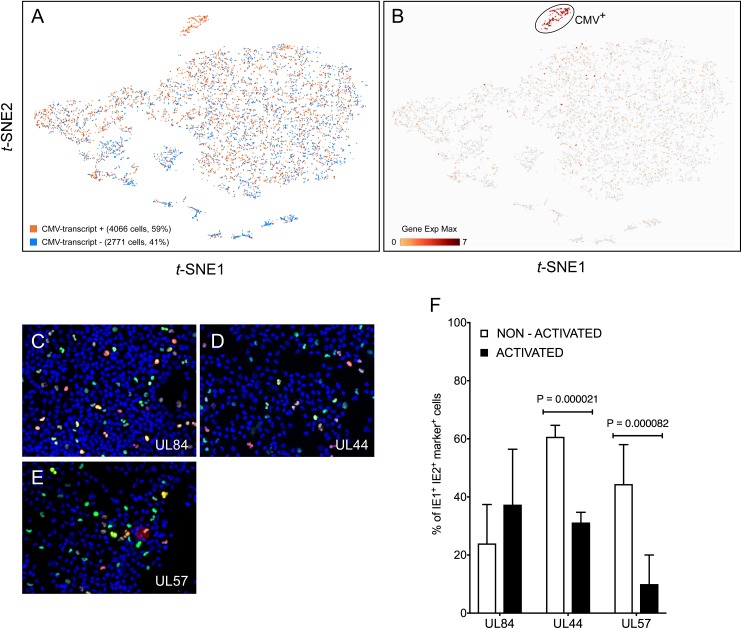
Detection of viral transcripts in a large proportion of cells in the population. **(A)** *t*-SNE projections of data from each of the 6,837 profiled activated cells depicted as dots colored based on their content in transcripts mapping to viral ORFs. **(B)** *t*-SNE projection of data colored based on their quantitative (Log_2_ Gene Exp Max) cumulative content in transcripts mapping to the viral ORFs UL122, UL123, UL112/113, UL84, TRS1, UL36, UL37, UL38, UL54, UL44, UL102, UL105, UL70, or UL57. **(C–E)** Merged representative images of activated myeloid cell populations infected with TB40/E at an MOI of 10, harvested at day 2 pi, and co-stained for IE1/IE2 (green) plus UL84 (red, **C**), or UL44 (red, **D**) or UL57 (red, **E**) and with Hoechst 33342 (blue) to visualize cell nuclei. Cells expressing IE1/IE2^+^ and the marker protein of interest appear yellow. **(F)** Percentage of IE1/IE2^+^ cells co-expressing the UL84, UL44, or UL57 proteins at day 2 pi, manually counted from 5 to 15 images/sample of non-activated and activated myeloid cells in seven independent experiments. Median, median absolute deviation and *P*-values from unpaired *T*-tests are shown.

Query of the *t*-SNE projection data for the presence of viral RNA revealed that 59% of the cells in the population contained at least one viral transcript (Figure 2A). RNAs mapping to the viral open reading frames (ORFs) UL4/UL5, US34, UL145, and UL16/17, and to the non-coding RNA2.7 and RNA1.2, were present in the largest proportions of cells (10, 6, 5, 5, 9, and 7%, respectively), accounting, together, for 30% of the CMV-transcript^+^ population. These viral RNA^+^ cells were dispersed throughout the entire population (Figure 2A), suggesting that infection had occurred in the majority of the cells. To avoid introducing perturbations potentially affecting cellular transcription, and as per standard protocols used in the vast majority of CMV tropism studies, cells were extensively washed in medium upon removal of the viral inoculum, but non-penetrated viral particles were not enzymatically removed. Consequently, some of the detected transcripts may have originated from virions still attached to the cell surface, or from penetrated capsids that did not reach the nucleus. The contribution provided by these particles to the detected pool of RNAs, however, was likely minimal because: (a) CMV capsids are unlikely to disassemble in the protease-free lysis buffer contained in the 10× Genomics Gel Bead-In-Emulsions; (b) the specific viral RNAs detected in the largest proportion of cells are not amongst those reported to be packaged into virions (Bresnahan and Shenk, 2000; Greijer et al., 2000; Goodrum et al., 2004), and (c) more than half of the 26 transcripts found in >200 cells mapped to viral ORFs known to be expressed with immediate-early or early kinetics (not shown), suggesting that they were newly synthesized from the viral genome.

To identify cellular factors potentially involved in restricting viral entry, the gene expression profile of CMV-transcript^+^ cells was compared to that of CMV-transcript^-^ cells. Only five cellular genes scored as differentially expressed between the two groups with *P* < 0.05, but none was present in the totality, nor in the majority, of CMV-transcript^-^ or CMV-transcript^+^ cells (Supplementary Dataset S1). The two genes expressed in the largest number of cells, RETN and TJP1 (for gene names, see Supplementary Table S1), were detected in only 303 and 124 cells, respectively, and were distributed in both populations: RETN was found in 153 CMV-transcript^+^ vs. 150 CMV-transcript^-^ cells, and TJP1 in 104 CMV-transcript^+^ vs. 20 CMV-transcript^-^ cells.

The extent of expression of genes encoding potential CMV entry receptors, such as EGFR (Wang et al., 2003; Chan et al., 2009; Buehler et al., 2016; Kim et al., 2017), PDGFRA (Soroceanu et al., 2008; Kabanova et al., 2016; Wu et al., 2017), THY1/CD90 (Li et al., 2015, 2016), the integrins αVβ3, α2β1, and α6β1 (Feire et al., 2004; Wang et al., 2005; Feire et al., 2010), and BSG (Vanarsdall et al., 2018) was also queried. EGFR, THY1/CD90, and integrins β3, α2, and α6 were either not expressed at all or were found in less than 10 cells, while PDGFRA was expressed in only 356 cells, and then only at low levels. Integrin β1 and BSG, by contrast, were present in larger numbers of cells (2568 and 5810, respectively), but these did not preferentially segregate with the CMV-transcript^+^ group.

Together, these findings indicate that viral entry is unlikely to be the main roadblock restricting infection onset, that cells devoid of viral transcripts do not transcribe specific factor(s) restricting virion entry, and that cells containing viral RNAs do not selectively express genes encoding entry facilitators, including surface molecules reported to act as CMV entry receptors in other cell types.

### Transcription of Viral Lytic Genes Proceeds in a Small Group of Cells Lacking Expression of Select Cellular Genes

Eleven genetic loci were identified as being required for efficient CMV genome replication in transient co-transfection replication assays (Pari and Anders, 1993; Pari, 2008). These encode the transcriptional activators/regulators IE1, IE2, UL112/113, UL84, and IRS1/TRS1, the anti-apoptotic factors UL36-38, and six members of the viral DNA replication complex, i.e., the DNA polymerase UL54, the polymerase accessory factor UL44, the helicase UL105, the primase UL70, the primase associated factor UL102, and the single-stranded DNA binding protein UL57. To identify cells ostensibly progressing toward lytic replication, the population was queried for the presence of viral transcripts encoding each of these proteins. A total of 278 cells, corresponding to 4% of the entire population and 7% of CMV-transcript^+^ cells were UL122^+^ (IE2), and/or UL123^+^ (IE1), and 42% of these expressed both. These proportions were in agreement with those obtained by immunofluorescence staining of infected cells from donor 113G harvested at day 1 pi (3.6% IE1/IE2^+^).

Consistent with progression toward lytic replication (Figure 1C), UL122^+^/UL123^+^ cells were also found to express transcripts encoding UL112/113 (91% triple-positive), UL84 (77%), IRS1/TRS1 (59%), UL36 (84%), UL37 (13%), UL38 (83%), and three replication complex components, namely UL54 (64%), UL105 (70%), and UL102 (56%) (Figure 2B). By contrast, RNAs corresponding to UL44, UL70, and UL57 were not detected. Staining of infected cells conducted at day 2 pi to allow sufficient time for protein synthesis, confirmed that the UL84 protein was present in 37 ± 19% of activated, IE1/IE2^+^ cells (Figure 2C,F). Interestingly, while presence of the UL44 and UL57 proteins was also observed (Figure 2D,E), the proportion of IE1/IE2^+^ cells co-expressing each of these gene products in activated cells was significantly lower than in non-activated cells (Figure 2F).

The data from cells containing the above viral transcripts, plus several others, comprised a tight and well separated cluster of 138 points on the *t*-SNE projection, which we collectively named CMV^+^ (Figure 2B). Comparison of the transcriptional profile of CMV^+^ and CMV^-^ cells identified 629 genes as being more highly expressed in CMV^-^ cells, but none was associated with significant *P*-values (<0.05), nor was present in the totality of CMV^-^ and absent in CMV^+^ cells. Sixty cellular genes had more than fourfold higher mean expression levels in CMV^-^ than in CMV^+^ cells, with nine being present in more than 50% of CMV^-^ cells but less than 50% of CMV^+^ cells (Supplementary Dataset S2 and Supplementary Figure S2). Encouragingly, and as expected for virus-exposed cells, five of these nine genes encoded well-known type I IFN-inducible proteins involved in mediating innate immune responses to viruses, i.e., MX1, OAS1, OAS2, IFIT3, and USP18. In line with activated myeloid cells, the majority of CMV^-^ cells also expressed the TNF-α and LPS-inducible protein CYTIP (Boehm et al., 2003), and the CD40L-inducible costimulatory molecule CD80 (Ranheim and Kipps, 1993), plus two genes, one coding for the orphan G protein-coupled receptor GPR157 and one for DUSP4, whose transcriptional regulation by viruses or other stimuli remains unassessed.

Together, these data indicate that although CMV transcripts are found in the majority of the population, lytic infection likely proceeds in only a small sub-group of cells containing lower amounts of a handful of genes, most of which encode known antiviral proteins.

### CMV^+^ Cells Are Closely Related to a Specific Sub-Cluster of Cells Within the Population

**FIGURE 3:**
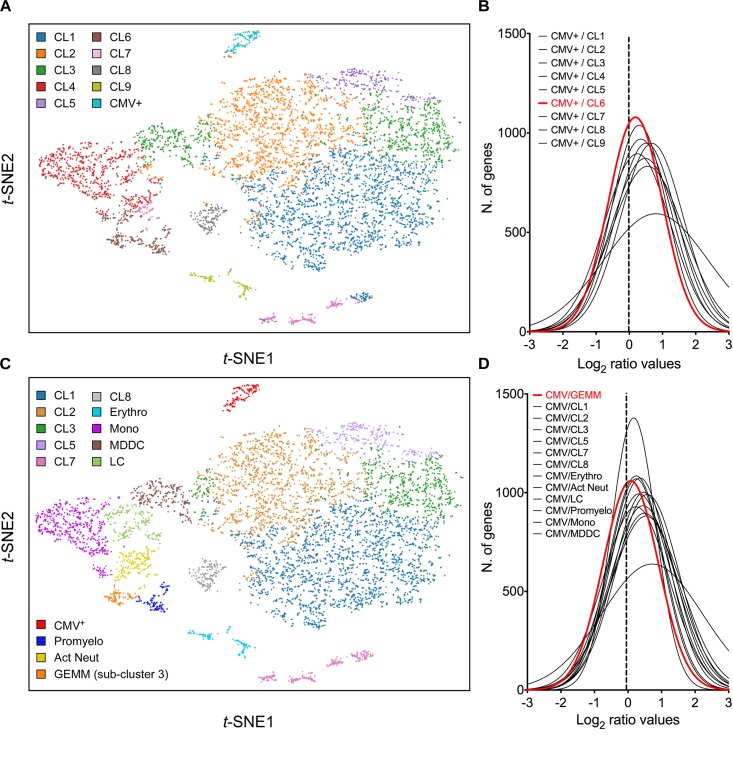
Identification of cluster 6 as the most closely related to the CMV^+^ cluster. **(A)** *t*-SNE projection of data from profiled activated cells partitioned into clusters by the *K*-means clustering algorithm using *K* = 10. **(B)** Distribution of Log_2_ ratio values obtained by dividing the mean number of transcripts/cell for each gene in the CMV^+^ cluster by the mean number of transcripts/cell for each gene in each of the other nine clusters. The distribution with the Log_2_ mean value closest to zero (dotted vertical line) is depicted by a red line. **(C)** *t*-SNE projection of data from profiled cells partitioned into clusters by the *K*-means clustering algorithm using *K* = 10, and further sub-divided based on marker gene expression. **(D)** Distribution of Log_2_ ratio values, as described in **(B)**, comparing the CMV^+^ cluster to the other 13 clusters. CL, cluster; Erythro, erythrocytes-megakaryocytes; Mono, monocytes; MDDC, monocyte-derived dendritic cells; LC, Langerhans cells; Promyelo, promyelocytes; Act Neut, activated neutrophils; GEMM, colony-forming unit-granulocyte, erythrocyte, monocyte/macrophage, megakaryocyte.

Cell clustering using the *K*-means algorithm revealed the presence of multiple different sub-groups of cells within the population, each characterized by distinct transcriptional profiles (Figure 3A). To identify the cluster most closely related to CMV^+^ cells, the mean number of transcripts/cell for each gene in the CMV^+^ cluster was divided by the mean number of transcripts/cell for each gene in each of the other nine clusters, and the frequency distribution of all Log_2_ ratios was plotted. A non-linear regression fit test using the least squares method revealed that all distributions were described by the Gaussian function, and that the histogram with the mean value closest to zero (0.184), the smallest standard deviation (0.814), and the highest *R*^2^ value (0.995) belonged to the CMV^+^ versus cluster 6 comparison (Figure 3B). A Wilcoxon signed rank test also identified the Log_2_ CMV^+^/cluster 6 ratio distribution as the one whose median values differed the least from zero, suggesting that the transcriptional profiles of CMV^+^ and cluster 6 cells were the most similar to each other.

To uncover the identity of cluster 6 cells, the genes most selectively expressed by this cluster relative to the rest of the population were identified using the 10× Genomics Cell Ranger software (CellRanger, 2018), and their expression range *in vivo* was assessed using publicly available gene expression databases and literature data. Sixty-nine genes were selected as being highly differentially expressed (Log_2_ cluster 6/rest of the cells fold change > 4, *P*-values < 10^-15^, Supplementary Dataset S3). Seven of these (ELANE, PRTN3, AZU1, MPO, PRSS57, CTSG, and RNASE2), coding for markers of neutrophil precursors (Mabbott et al., 2013; Rapin et al., 2014) were predominantly or exclusively expressed in a sub-group of 116 cells, which we designated “promyelocytes” (Supplementary Figure S3A). Four more genes (RETN, S100A8, S100A9, and S100A12), encoding proteins secreted by activated neutrophils under pro-inflammatory conditions (Bostrom et al., 2009; Tardif et al., 2015), were abundant in promyelocytes and in a separate group of ∼ 205 cells, designated “activated neutrophils” (Supplementary Figure S3B). The remaining 57 genes encoded mostly DNA replication and cell cycle regulators, and were present, either exclusively or overlapping with promyelocytes and CMV^+^ cells, in a third group of 93 cells (Supplementary Figure S3C), designated “sub-cluster 3.”

In addition to separating cluster 6 into three sub-clusters, other groups of cells were identified based on their expression of known markers such as CD14 and CD68 (monocytes), CD207/langerin and CD1a (Langerhans cells), CD1b (monocyte-derived dendritic cells), and hemoglobins (erythrocytes) (Figure 3B). Of note, and in keeping with CD34^+^ HSC differentiation toward myeloid (rather than lymphoid) lineages, no T or B cell specific transcripts were found.

While none of the promyelocyte- and activated neutrophil-specific genes were also expressed by CMV^+^ cells, 18 (32%) of sub-cluster 3 marker genes were shared, some almost exclusively, with the CMV^+^ group. This suggested that sub-cluster 3 cells in specific might be related to the CMV^+^ cell cluster. To further verify this, the mean number of transcripts/cell for each gene in the CMV^+^ cluster was divided by the mean number of transcripts/cell for each gene in each of the other 13 clusters, and the frequency distribution of Log_2_ ratios was plotted. The histogram whose median value differed the least from zero did indeed correspond to the CMV^+^ versus sub-cluster 3 comparison (Figure 3D), confirming that the transcriptional profile of these two groups are the most closely related.

Sub-cluster 3 also contained the highest proportion of viral transcript^+^ cells (74%), followed by Langerhans cells (72%), monocytes (68%), cluster 2 (65%), monocyte-derived dendritic cells (64%), cluster 5 (62%), cluster 3 (59%), cluster 1 (57%), promyelocytes (42%), erythrocytes (41%), activated neutrophils (35%), cluster 8 (34%), and cluster 7 cells (32%).

### Sub-Cluster 3 Is Comprised of Cells With CFU-GEMM Hallmarks

To more precisely identify the cell type comprising sub-cluster 3, the list of 115 genes more abundantly (average transcript count > 0.3) and most differentially (Log_2_ fold change > 3, *P* < 0.0005) transcribed in these cells relative to all other clusters was compared to gene lists from two recently published single-cell analyses of human hematopoiesis (Velten et al., 2017; Karamitros et al., 2018) (Supplementary Dataset S4). Seventy-two transcripts were found among the list of genes reported to be differentially expressed in 16 discrete bone marrow populations by Velten et al. (2017), with the largest proportion falling within the “G2/M phase” (56%) and the “Immature myeloid progenitors with high cell cycle activity” (24%) categories. A total of 108 genes were also found among the transcripts classified as differentially expressed in seven human cord blood populations by Karamitros et al. (2018), with the vast majority belonging to the “common myeloid progenitor” population (88%), followed by the megakaryocyte/erythroid progenitor compartment (6%). This suggested that sub-cluster 3 cells might consist of multipotent progenitors which, in contrast to HSC, are known to be highly proliferative and metabolically active (Cabezas-Wallscheid et al., 2014; Laurenti and Gottgens, 2018).

Within the CMV^+^ population, half of the 115 abundantly/differentially expressed genes were almost exclusively associated with sub-cluster 3, followed by shared expression with promyelocytes, erythrocytes/megakaryocytes, activated neutrophils, and monocytes (Supplementary Dataset S4). Thirty-six of these genes were also expressed in CMV^+^ cells, with the majority being shared with the promyelocytes and erythrocytes/megakaryocytes clusters. Together, these data indicate that sub-cluster 3 is comprised of proliferating cells expressing erythroid, monocytic and granulocytic markers, which we surmised might represent CFU-GEMM oligopotent progenitors.

**FIGURE 4:**
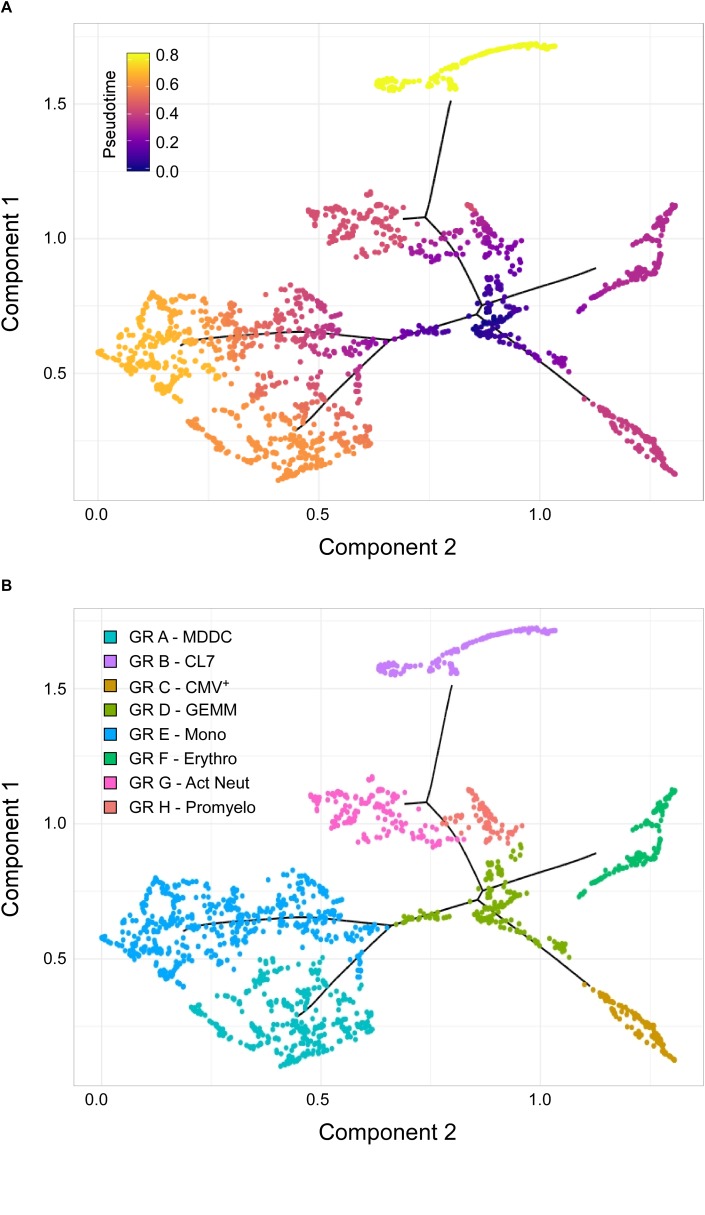
Identification of sub-cluster 3 as the origin of promyelocytes, activated neutrophils, erythrocytes, megakaryocytes, monocytes, and CMV^+^ cells. **(A)** Pseudotime ordering of data from cells belonging to the CL7, erythro, mono, MDDC, CMV, promyelo, act neut and sub-cluster 3 groups shown in Figure 3C into a two-dimensional component space using Monocle. The main path of the minimum spanning tree is depicted by solid black lines arising from a central root of cells with a pseudotime of zero (dark blue dots), and branching outward to clusters with higher pseudotime values, representing differentiated cell types (purple, orange, and yellow dots). **(B)** Cell group labeling based on the expression of key marker genes identified with Seurat. GR, group; MDDC, monocyte-derived dendritic cells; CL7, CL7 from Figure 3C; GEMM, colony-forming unit-granulocyte, erythrocyte, monocyte/macrophage, megakaryocyte; Mono, monocytes; Erythro, erythrocytes; Act Neut, activated neutrophils; Promyelo, promyelocytes.

To further test this hypothesis, cells belonging to the cluster 7, erythro, mono, MDDC, CMV^+^, promyelo, act neut and sub-cluster 3 groups depicted in Figure 3C were ordered along trajectories corresponding to their inferred differentiation pathways using Monocle (Trapnell et al., 2014). A trajectory with four main branches extending from a rooted center was generated (Figure 4A), and the identity of cells composing each of the eight groups was uncovered using Seurat (Butler et al., 2018) (Figure 4B and Supplementary Figure S4). Cells in group D, the root center, expressed the same key genes as sub-cluster 3 cells in Figure 3C, while its closest neighbors, group E, F, G, and H, expressed markers typical of the monocytes, erythrocytes, promyelocytes and activated neutrophils clusters in Figure 3C, respectively (Supplementary Figure S4 and Supplementary Dataset S5). The most isolated cluster of cells, group B, was related to CL7 in Figure 3C, and differentially expressed CD52 and FCER1A (Supplementary Dataset S5).

Thus, the observed pseudotime distances and cluster organization strongly implicated group D as the most likely origin of CMV^+^ cells, as well as of erythrocytes, megakaryocytes, promyelocytes, neutrophils, and monocytes, suggesting that cells comprising group D/sub-cluster 3 might indeed represent CFU-GEMM oligopotent progenitors.

### Expression of Genes With Functions in Energy Production, Cell Cycle Control, RNA and Protein Metabolism Is Higher in Both GEMM and CMV^+^ Cells

To understand why cells in sub-cluster 3 (relabeled GEMM) were the most closely related to CMV^+^ cells, we sought to identify which set of genes and, consequently, which cellular functions, were most differentially regulated in GEMM and CMV^+^ cells with respect to the rest of the population. A set of 1989 genes was identified by the 10× Genomics Cell Ranger software as being more selectively expressed in the CMV^+^ and GEMM clusters relative to all other clusters. The majority of these genes (1361, or 68% for GEMM, and 1460, or 73% for CMV^+^ cells) were associated with positive Log_2_ fold change values, indicating that most of the GEMM-specific genes were more highly expressed in these cells than elsewhere, and that infection was accompanied by a strong transcriptional up-regulation of cellular genes (Supplementary Dataset S6, Sheet S1).

The differentially expressed genes were then partitioned between “synchronous” and “asynchronous,” depending on whether their transcription was similarly regulated in GEMM and CMV^+^ cells or not. Genes that were up-regulated in GEMM cells relative to the rest of the population, and that were expressed to similar levels or further up-regulated in CMV^+^ cells (total = 1077), as well as genes that were down-regulated in GEMM cells and that were expressed to similar levels or further down-regulated in CMV^+^ cells (total = 325) were considered synchronous, while genes that were up-regulated in GEMM but down-regulated at least two-fold in CMV^+^ cells, and vice-versa, were labeled asynchronous (total = 587). The majority (1402, 70%) of the selected genes fell into the synchronous category. Of these, most were up-regulated in the GEMM cluster and expressed to similar levels in CMV^+^ cells, with only 28 genes being further induced in infected cells, suggesting that GEMM cells already contain large numbers of transcripts beneficial (or neutral) to infection (Supplementary Dataset S6, Sheet S1). As levels of down-regulated genes were also mostly maintained without any further repression by infection, we hypothesized that GEMM cells might be preferentially infected because their transcriptional landscape requires the least amount of optimization by viral effectors.

**FIGURE 5:**
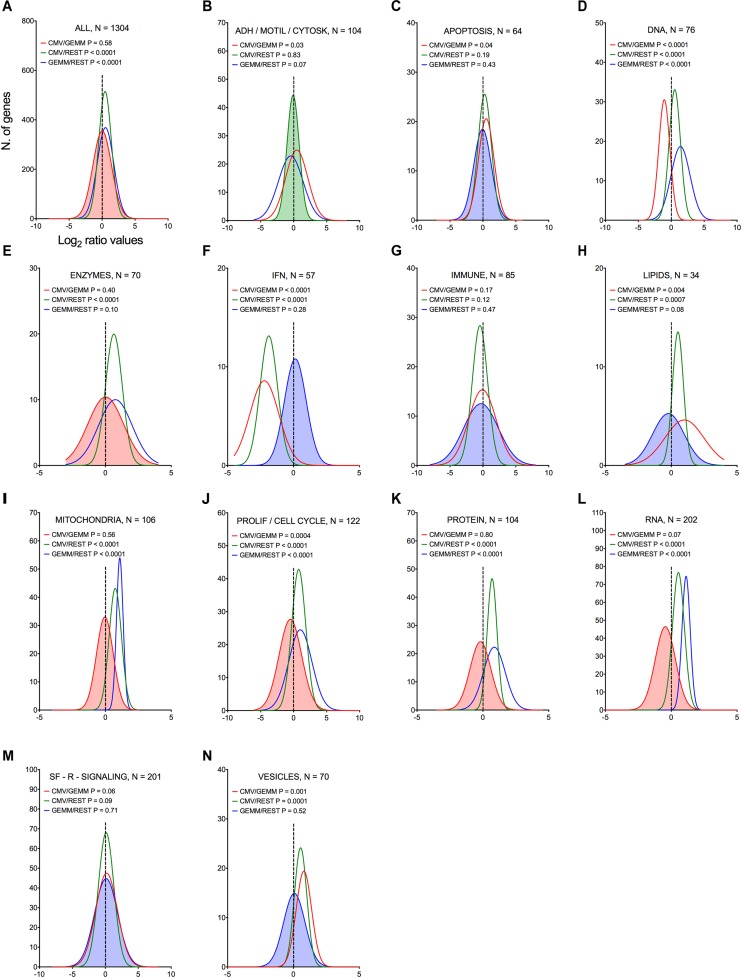
Differential expression of genes belonging to multiple functional categories in GEMM cells, CMV^+^ cells, and in the rest of the population. Log_2_ ratio value distributions obtained by dividing the mean number of transcripts/cell for each gene in the CMV^+^ or GEMM clusters by the mean number of transcripts/cell in the rest of the cells (CMV/REST, green line, and GEMM/REST, blue line) or in GEMM cells (CMV/GEMM, red line). The distribution obtained from all genes is shown in **(A)**, while **(B–N)** show the distributions of genes falling in each functional category. The Wilcoxon signed rank test was used to identify populations with median values significantly different from zero. The population with the lowest *P*-value is highlighted by coloring of the area under the curve. The dashed line marks the ratio = 1 point. N, number of genes in each category; ADH/MOTIL/CYTOSK, adhesion/motility/cytoskeleton; IFN, interferon; PROLIF/CELL CYCLE, proliferation/cell cycle; SF-R-SIGNALING, soluble factors/receptors/signaling.

To pinpoint the functional areas distinguishing GEMM cells from the remainder of the population, the most differentially expressed synchronous genes, and all of the asynchronous genes (1304 in total) were partitioned into 15 categories based on their encoded functions (Supplementary Dataset S6, Sheet S2). The transcript abundance of each gene found in the CMV^+^ or GEMM clusters was then divided by the abundance in the rest of the cells (CMV/REST and GEMM/REST) or in GEMM cells (CMV/GEMM), and the distributions of the Log_2_ ratio values were plotted (Figure 5). As expected, only the GEMM/REST and CMV/REST, but not the CMV/GEMM ratio distributions of all genes were identified by the Wilcoxon signed rank test as having Log_2_ median values significantly different from zero (Figure 5A). Genes with roles in mitochondrial functions (Figure 5I), proliferation and cell cycle control (Figure 5J), RNA metabolism (Figure 5L) and protein processing (Figure 5K) were also more highly expressed in both GEMM and CMV^+^ cells, and were thus further scrutinized.

#### Mitochondria

**FIGURE 6:**
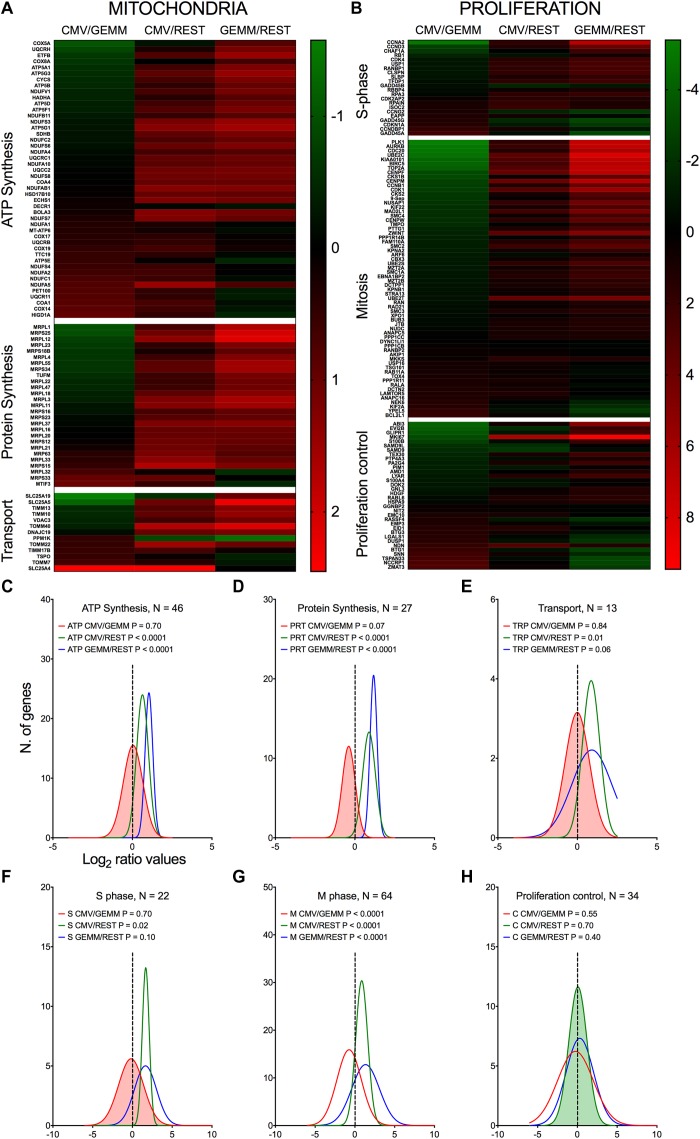
Differential expression of genes with roles in mitochondrial function and proliferation control in GEMM cells, CMV^+^ cells, and the rest of the population. Heatmap **(A,B)** and distributions **(C–H)** of Log_2_ ratio values obtained by dividing the mean number of transcripts/cell of genes with roles in mitochondrial functions **(A,C–E)** and in proliferation control **(B,F–H)** as found in the CMV^+^ or GEMM clusters by the mean number of transcripts/cell in the rest of the cells (CMV/REST, green line, and GEMM/REST, blue line) or in GEMM cells (CMV/GEMM, red line). The heatmap color scales refer to the Log_2_ ratio values. The Wilcoxon signed rank test was used to identify populations with median values significantly different from zero. The population with the lowest *P*-value is highlighted by coloring of the area under the curve. The dashed line marks the ratio = 1 point. N, number of genes in each category.

Genes involved in ATP production, mitochondrial protein synthesis, and mitochondrial transport were consistently more abundant in GEMM cells than elsewhere (Figure 6A,C–E, blue lines), with their expression levels remaining largely unchanged in CMV^+^ cells (Figure 6A,C–E, red lines). Among these, genes encoding members of the ATP synthase and NADH dehydrogenase complexes of the electron transfer chain were the most represented, together with genes encoding mitochondrial ribosomal proteins (Supplementary Dataset S6, Sheet S3). This suggests that infection might preferentially start in GEMM cells due to the existence of an intracellular environment already geared toward high energy production, and hence capable of supporting the large metabolic requirements of viral replication. We did indeed previously observe a similarly strong up-regulation of genes with functions in oxidative phosphorylation and fatty acid β-oxidation in infected fibroblasts at late times pi (Hertel and Mocarski, 2004), indicating that the enhancement of mitochondrial functions is a key feature of infection, shared by different cell types.

#### Proliferation/Cell Cycle

Consistent with the notion that multipotent progenitors are highly proliferative (Cabezas-Wallscheid et al., 2014; Laurenti and Gottgens, 2018), GEMM cells expressed higher levels of genes encoding S and M phase effectors than the rest of the population (Figure 6B,F,G, blue lines and Supplementary Dataset S6, Sheet S4). CMV infection of fibroblasts was reported by us and others to repress expression of genes promoting entry into S phase, while simultaneously inducing expression of DNA synthesis effectors (Bresnahan et al., 1996; Lu and Shenk, 1996; Dittmer and Mocarski, 1997; Hertel and Mocarski, 2004). In keeping with these observations, CMV^+^ cells contained lower transcript amounts of genes promoting entry into S phase, such as CCNA2, CCND3, MKI67, and RB1, but higher transcript levels of genes encoding inhibitors of S phase progression, including BTG1, BTG3, CCNDBP1, CDKN1A, and the HSC quiescence-promoting gene NDN (Asai et al., 2012). Transcription of DNA replication effectors was, by contrast, inconsistently induced. While expression of some genes, such as the catalytic subunit of the DNA polymerase delta (POLD2) and its interacting protein POLDIP2, RPA3, and RPAIN, was high, transcription of others such as PCNA, MCM3, MCM7, and FEN1 was reduced in CMV^+^ cells. We speculate that this mixed transcriptional regulation might be typical of the early phase of infection, when viral factors are still in the process of gaining control over cell proliferation, while at later times, when data from fibroblasts were collected (Hertel and Mocarski, 2004), viral DNA synthesis is already fully established.

We previously reported that CMV infection induces the appearance of aberrant mitotic figures, supported by the induction of numerous genes involved in M phase progression (Hertel and Mocarski, 2004). Although this feature was shared by different CMV strains, it was by far most evident with the attenuated strain AD169 than with TB40/E (Hertel et al., 2007). Consistent with the TB40/E pattern, only a minority of the 63 genes with functions in mitosis were maintained to high levels in CMV^+^ cells, while the rest were down-regulated (Figure 6B,G), including the two main components of the mitosis-promoting factor, CDK1 and CCNB1, chromatin condensation agents (SMC2, SMC4, ZWINT, and TOP2A), mitotic spindle assembly controllers (AURKB, BIRC5, PLK1, MAD2L1, and CENPF), components of the anaphase-promoting complex (CDC20 and PTTG1), and cytokinesis effectors (SEPT9, ARF6, and RAB11A).

Together, these data are consistent with a CMV-induced block in cell proliferation, aimed at curtailing usage of cellular resources for processes irrelevant to viral replication, such as mitosis, and steering others, such as those devoted to cellular genome replication, toward viral DNA production instead.

#### RNA Metabolism

**FIGURE 7:**
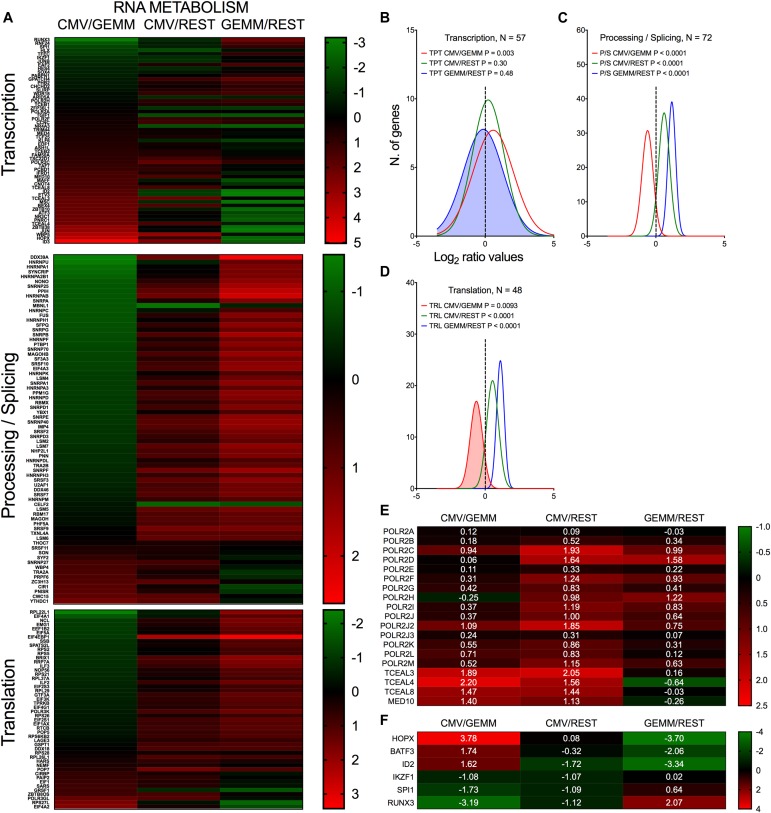
Differential expression of genes with roles in RNA metabolism in GEMM cells, CMV^+^ cells, and the rest of the population. Heatmap **(A,E,F)** and distributions **(B–D)** of Log_2_ ratio values obtained by dividing the mean number of transcripts/cell of genes with roles in RNA transcription, processing and translation as found in the CMV^+^ or GEMM clusters by the mean number of transcripts/cell in the rest of the cells (CMV/REST, green line, and GEMM/REST, blue line) or in GEMM cells (CMV/GEMM, red line). The heatmap color scales refer to the Log_2_ ratio values. Numbers in white font in **(E,F)** report the Log_2_ ratio values of each gene. The Wilcoxon signed rank test was used to identify populations with median values significantly different from zero. The population with the lowest *P*-value is highlighted by coloring of the area under the curve. The dashed line marks the ratio = 1 point. N, number of genes in each category.

As expected for metabolically active cells, expression of numerous genes involved in RNA processing, splicing and translation were more highly expressed in GEMM and in CMV^+^ cells than in the rest of the population (Figure 7A,C,D, blue and green lines and Supplementary Dataset S6, Sheet S5). By contrast, expression of ∼70% of transcription-related genes was similar in GEMM and in the rest of the cells, but was up-regulated in CMV^+^ cells (Figure 7A,B, blue and red lines). Particularly revealing of the strong impetus of infection toward stimulating cellular gene transcription on a broad scale was the induction of several RNA polymerase II subunits and elongation factors (Figure 7E), while among transcription factors, alterations in expression of several genes with essential roles in hematopoietic development was especially interesting (Figure 7F). These include HOPX, which regulates primitive hematopoiesis (Palpant et al., 2017), BATF3, vital for the development of conventional cross-presenting CD8α^+^ dendritic cells, ID2, whose expression in CD34^+^ HSC inhibits the development of dendritic cell precursors (Ling et al., 2014), RUNX3, whose depletion leads to defects in the proliferation and differentiation of activated cytotoxic CD8^+^ T cells, helper Th1 cells and NK cells, and to the disappearance of skin Langerhans cells (Lotem et al., 2017), IKZF1, essential for normal lymphopoiesis and for myeloid, megakaryocyte and erythroid differentiation (Marke et al., 2018), and SPI1/PU.1, which is critical for the generation of all hematopoietic lineages (Burda et al., 2010). Should it also happen *in vivo*, dysregulation of these genes’ expression may powerfully affect the development and function of multiple arms of the hematopoietic system, potentially contributing to the well-known problems related to CMV infection in fetuses and in hematopoietic stem cell transplantation settings.

Thus, the early stages of infection appear to be associated with a sharp push toward increased production of RNA synthesis and processing effectors, which are likely required to support viral gene transcription in order to fine-tune viral control over a variety of cellular processes, including cell differentiation.

#### Protein Metabolism

**FIGURE 8:**
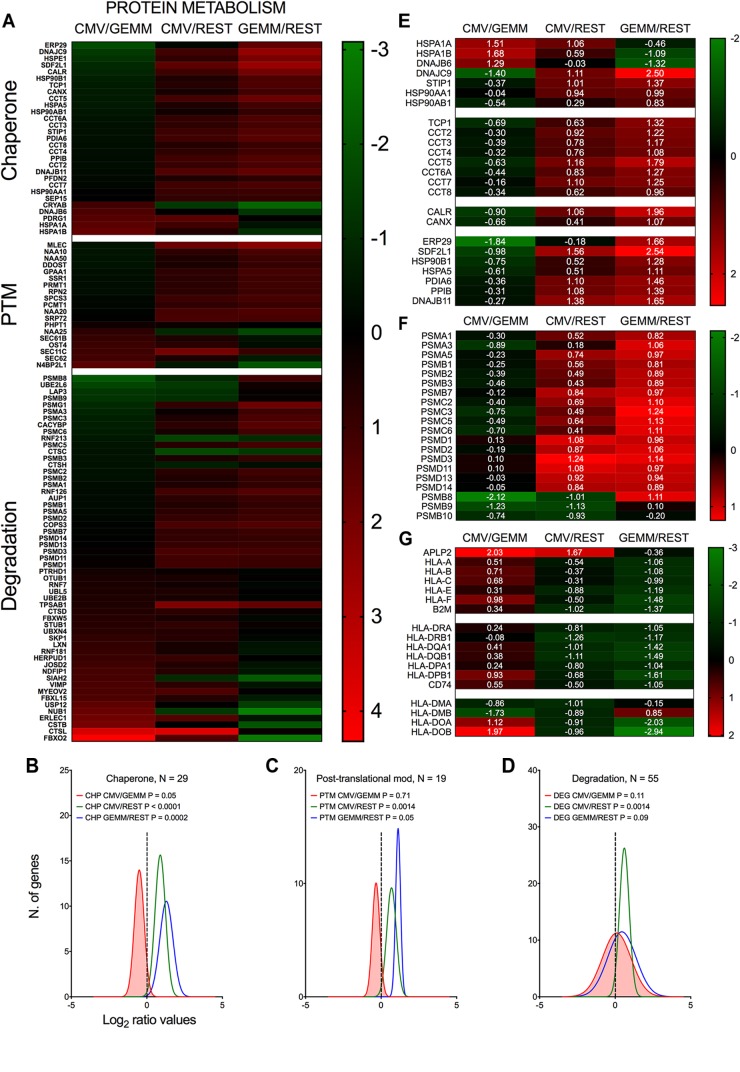
Differential expression of genes with roles in protein metabolism and antigen presentation in GEMM cells, CMV^+^ cells, and the rest of the population. Heatmap **(A,E,F,G)** and distributions **(B–D)** of Log_2_ ratio values obtained by dividing the mean number of transcripts/cell of genes with roles in protein metabolism as found in the CMV^+^ or GEMM clusters by the mean number of transcripts/cell in the rest of the cells (CMV/REST, green line, and GEMM/REST, blue line) or in GEMM cells (CMV/GEMM, red line). The heatmap color scales refer to the Log_2_ ratio values. Numbers in white font in E-G report the Log_2_ ratio values of each gene. The Wilcoxon signed rank test was used to identify populations with median values significantly different from zero. The population with the lowest *P*-value is highlighted by coloring of the area under the curve. The dashed line marks the ratio = 1 point. N, number of genes in each category.

In keeping with the robust infection-associated stimulation of gene translation, expression of numerous protein chaperones and post-translational modifiers was also higher in both GEMM and CMV^+^ cells than in the rest of the population (Figure 8A–C, blue and green lines and Supplementary Dataset S6, Sheet S6). Chaperone-assisted protein folding occurs via three main routes, the simplest one being via interactions with single HSP70 or HSP90 family members. Some polypeptides require the sequential binding of HSP70 and HPSP90 instead, while others need the intervention of the chaperonin containing TCP1 complex (CCT) (Kabir et al., 2011). Both HSP70 coding transcripts, HSPA1A and HSPA1B, and their co-chaperone DNAJB6 were expressed to lower levels in GEMM cells than in the rest of the population, and were up-regulated in infected cells. The adaptor protein STIP1, which coordinates protein transfer from HSP70 to HSP90, the inducible (HSP90AA1) and constitutive (HSP90AB1) HSP90 isoforms, and all eight subunits of the CCT complex were expressed at higher levels in both GEMM and CMV^+^ cells. A similar pattern of regulation was observed for calnexin (CANX) and calreticulin (CALR), and for seven out of eleven members of the large endoplasmic reticulum (ER)-localized multiprotein complex (HSPA5, DNAJB11, HSP90B1, PPIB, PDIA6, SDF2L1, and ERP29), which, together, comprise the ER protein quality control system (Meunier et al., 2002; Williams, 2006) (Figure 8E).

Levels of numerous genes with roles in protein degradation were also slightly higher in GEMM and CMV^+^ cells (Figure 8A,D, blue and green lines). Of particular interest was the up-regulation of 17 subunits (out of 33) of the proteasome (Figure 8F). Protein degradation may benefit the virus by removing unwanted cellular polypeptides and damaged or misfolded proteins, while simultaneously enhancing amino acid availability. An essential role of the proteasome, however, is to produce antigenic peptides suitable for presentation on major histocompatibility complex (MHC) class I molecules, an activity extremely detrimental to virus spread. In the immunoproteasome, the proteolytic subunits PSMB5, 6 and 7 are replaced with PSMB8, 9 and 10. Very intriguingly, and consistent with data from infected fibroblasts (Khan et al., 2004), expression of these latter subunits was down-regulated in CMV^+^ cells (Figure 8F).

In addition to curtailing the ability of the immunoproteasome to produce antigenic peptides, MHC class I activities were also negatively impacted by the strong transcriptional induction of APLP2, an enhancer of MHC class I internalization and turnover (Tuli et al., 2008a,b). Rather intriguingly, transcript levels of genes encoding the three main MHC class I molecules, HLA-A, -B, and –C, and of their binding partner B2M, as well as of the three main MHC class II isotypes, HLA-DR, -DQ, and -DP and the invariant chain CD74 were already ∼2.5-fold lower in GEMM cells than in the rest of the population and were not further reduced in CMV^+^ cells (Figure 8G). By contrast, expression of HLA-DMA and HLA-DMB, which assist in the binding of high affinity antigenic peptides into MHC class II (ten Broeke et al., 2013), were repressed while transcription of HLA-DOA and HLA-DOB, which increase tolerance to self-peptides (ten Broeke et al., 2013), was increased (Figure 8G).

Together, these data underscore the strong effects of infection on fine-tuning the cellular protein “portfolio” to match the virus’ needs, and highlight the selectivity of viral effectors in modulating the expression of specific cellular proteins in order to protect infected cells from detection and elimination by the host immune system.

### Expression of Genes With Functions in IFN-Mediated Antiviral Defenses Is Similar in GEMM and in the Rest of the Cells, but Is Strongly Down-Regulated in CMV^+^ Cells

Akin to genes belonging to categories of apoptosis, immune, lipids, soluble factors/receptors/signaling and vesicles (Figure 5C,G,H,M,N, blue line), transcript levels of IFN-related genes were overall similar in GEMM cells and in the rest of the population (Figure 5F, blue line). Very excitingly, however, this category contained the most strongly down-regulated genes of all in CMV^+^ cells (median Log_2_ CMV/GEMM ratio value of –1.9, *P* < 0.0001, Figure 5F, red line).

**FIGURE 9:**
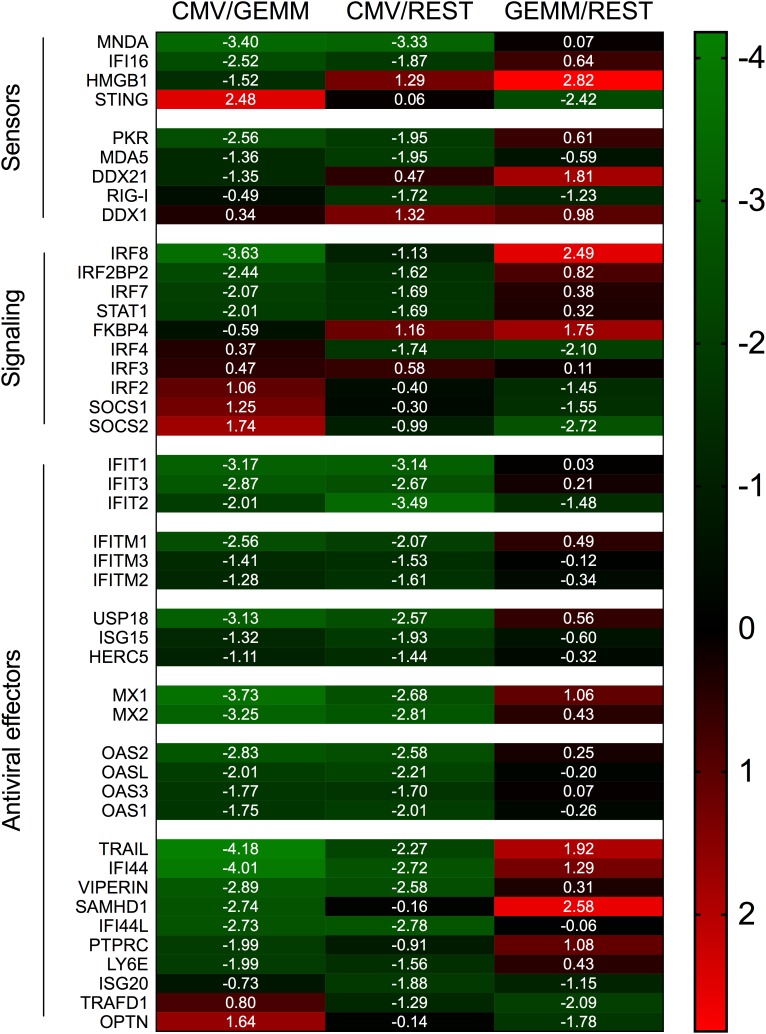
Differential expression of IFN-related genes in GEMM cells, CMV^+^ cells, and the rest of the population. Heatmap of Log_2_ ratio values obtained by dividing the mean number of transcripts/cell of IFN-related genes as found in the CMV^+^ or GEMM clusters by the mean number of transcripts/cell in the rest of the population (CMV/REST and GEMM/REST) or in GEMM cells (CMV/GEMM). The heatmap color scale refers to the Log_2_ ratio values. Numbers in white font report the Log_2_ ratio values of each gene.

Compared to the rest of the population, GEMM cells contained higher levels (median ratio, ∼1.5-fold) of transcripts encoding sensors of viral double-stranded DNA and RNA, such as IFI16 (Unterholzner et al., 2010), HMGB1 (Yanai et al., 2009), DDX58/RIG-I (Pichlmair et al., 2006), IFIH1/MDA5 (Kato et al., 2006) and EIF2AK2/PKR (Meurs et al., 1990), of signaling mediators like STAT1, and of transcriptional activators such as IRF3, IRF7, and IRF8 (Zhao et al., 2015), but lower levels (median ratio, ∼4-fold) of negative regulators of IFN production and signaling such as IRF2 (Negishi et al., 2005), IRF4 (Harada et al., 1989), TRAFD1 (Sanada et al., 2008), and SOCS1 (Yoshimura et al., 2018). Expression of IFN effectors including IFIT1, IFIT2, and IFIT3, which recognize and prevent translation of virally produced triphosphorylated RNA molecules (Vladimer et al., 2014), IFITM1, IFITM2, and IFITM3, which block infection at multiple steps including entry (Shi et al., 2017), ISG15 and its conjugating (HERC5) and de-conjugating (USP18) enzymes, which disrupt the activity of viral proteins by ISGylation (Perng and Lenschow, 2018), as well as of known (MX1, MX2, OAS1, OAS2, OAS3, and OASL) (Hovanessian and Justesen, 2007; Haller et al., 2015), or suspected anti-viral proteins such as viperin (Helbig and Beard, 2014), SAMHD1 (Li et al., 2017), and ISG20 (Zheng et al., 2017) were instead similarly abundant in GEMM and the rest of the cells (median ratio, ∼1.1-fold) (Figure 9).

Together, these data suggest that GEMM cells are not defective in their ability to detect, respond and potentially antagonize viral infection. Rather, GEMM cells appear to be similarly, or even more responsive than the rest of the population, indicating that the lack of appropriate cellular defenses is unlikely to be the main reason for their preferential infection. Interestingly, and similar to the situation with MHC class I and II genes, transcriptional modulation of these genes in CMV^+^ cells appeared to be selective: while mRNA levels of most IFN antiviral effectors were powerfully reduced, transcription of negative regulators was enhanced, with the notable exception of the adaptor protein TMEM173/STING (Ishikawa and Barber, 2008; Zhong et al., 2008; Ishikawa et al., 2009; Tanaka and Chen, 2012) and the TBK1 activator OPTN (Outlioua et al., 2018), which are both involved in IFN production following CMV DNA detection by MB21D1/cGAS (Paijo et al., 2016). Taken together, these findings provide support to our theory whereby infection preferentially begins in GEMM cells due to their higher metabolic, proliferative, and RNA and protein synthesis rates, rather than to impairments in their capacity to mount strong cellular defenses.

## Discussion

CD34^+^ HSC-derived myeloid cells are semi-permissive to CMV lytic infection, with progeny being produced (Figure 1C) despite the occurrence of viral gene expression in only a small fraction of cells (Figure 1A,B). While the proportion of cells supporting infection onset is increased by activation, viral yields are reduced, suggesting that resistance to infection in these cells is multilayered and can impact more than one steps in the viral life cycle (Hertel et al., 2003; Lauron et al., 2014; Coronel et al., 2015, 2016).

Here, we sought to deepen our understanding of CMV tropism determinants by correlating specific cell type(s) and cellular pathways with CMV gene expression upon infection of a population of activated myeloid cells derived from the HSC of a single cord blood donor, selected as representative. As only one population was profiled, the collected data may not be common to *all* cell cultures. Nevertheless, since data collected from donor 113G-derived cells are consistent with the median values derived from all the populations tested (Figure 1, red and blue lines), we believe that our findings are illustrative. While profiling of activated and non-activated cells derived from additional HSC donors and at multiple times pi is currently planned, the dataset we supply in this work provides the first opportunity for investigators to analyze cellular gene expression changes occurring in CMV^+^ and viral transcript^low^ or transcript^-^ cells co-existing within the same population of myeloid cells. As such, our dataset represents a new, unique and valuable resource.

While in homogeneous and permissive cell populations (such as fibroblasts) the probability for a cell to remain free of viral particles at an MOI of ten is null, our data show that ∼40% of activated myeloid cells do not contain any viral RNAs at day 1 pi (Figure 2A). Although this may depend, at least in part, on timing and detection limits, we speculated that this portion of the population might be more resistant to viral entry due to the presence of specific restriction factors and/or the absence of entry facilitators. However, no specific cellular genes were identified as being selectively transcribed in viral RNA^+^ or RNA^-^ cells, including transcripts coding for proteins currently known to support virion entry. Because our myeloid cell cultures are highly heterogeneous (Figure 3), preferential infection of select sub-groups may still have been facilitated by the expression of specific genes. BSG, for instance, was present in 99% of GEMM cells but in only 60% of cluster 7 cells. Conversely, viral RNA^-^ cells may have resisted infection owing to the expression of subset-specific molecules. Yet, the fact that no “universal” entry resistance/enabling gene(s), expressed by all viral transcript^+/-^ cells, could be identified implies that such gene(s) may not exist. This is in contrast to other cell types such as endothelial and epithelial cells, whose infection instead depends on the expression of surface molecules (such as BSG), acting as receptors for specific glycoprotein complexes present on the virion’s surface (Vanarsdall et al., 2018).

Only a small fraction (∼3%) of CMV-transcript^+^ cells expressed multiple viral ORFs at high levels, including those encoding factors required for efficient viral genome replication (CMV^+^ cluster in Figure 2B). This suggests that these cells might be the ones progressing toward lytic replication, an assumption corroborated by the presence, in a similar proportion of cells (Figure 2C–F), of viral proteins involved in viral DNA replication (UL84, UL44, and UL57), and by the production of viral progeny (Figure 1C). CMV^+^ cells may, alternatively, be abortively infected, with viral progeny being produced by another set of cells not yet expressing viral replication markers at day 1 and 2 pi. This alternative hypothesis, however, appears rather implausible. Interestingly, a similar scenario was recently encountered following single-cell RNA sequencing of TB40/E-infected CD14^+^ cells (Shnayder et al., 2018). Elevated levels of viral transcripts were observed in just ∼2% of monocytes, while the rest of the population, which contained lower amounts of a wide range of viral transcripts, were interpreted as being latently infected. This led us to speculate that CMV-transcript^+^ cells not belonging to the CMV^+^ cluster might be latently infected, or on a path toward latency. While this hypothesis requires additional testing, it remains a thrilling possibility, especially in view of recently presented evidence supporting the potential association of viral latency with quantitative rather than qualitative changes in viral gene expression (Shnayder et al., 2018).

Although expression of CD207/langerin and CD1a was observed in multiple cells, Langerhans cells did not appear to be the main source of CMV^+^ cells. Rather, multiple lines of evidence indicate that CMV^+^ cells derive from a cluster with the hallmarks of GEMM progenitors, albeit devoid of transcripts coding for some of the markers traditionally used to describe this population, i.e., CD34, CD38, and CD123. As none of the genes we and others (Velten et al., 2017; Karamitros et al., 2018) found to be selectively expressed by these cells encode surface molecules, their isolation from myeloid populations differentiated *in vitro* or from hematopoietic tissues is particularly challenging. Consequently, we do not currently have direct evidence that this specific cell type can support CMV lytic infection *in vivo*. Very recent data from single-cell RNA-seq analyses of hematopoietic processes have revealed that lineage development is a continuous process, more usefully depicted by Waddington’s landscapes (Waddington, 1957), than by more rigid cell differentiation trees. In this emergent scenario, CD34^+^ HSC are visualized as beads rolling along a surface stretching from a higher to a lower point in space, and containing ridges and valleys. These ridges, corresponding to barriers separating individual lineages, are smaller near the top and become increasingly higher toward the bottom, as expression of fate mediators progresses in each cell. Once ridges become too high, cells can no longer change their identity, and terminal lineages are established (Velten et al., 2017; Buenrostro et al., 2018; Karamitros et al., 2018; Povinelli et al., 2018). We believe that the permissive cell type we identified in this study corresponds to a mid-point along this surface, characterized by the loss of pluripotency, but not yet enclosed by the high ridges separating granulocytes, monocytes, erythrocytes, and megakaryocytes from each other. While this cell type is more than likely to exist *in vivo*, it may have been missed in previous studies of CMV tropism due to its rarity, and/or to the lack of specific surface markers. Determining if blood-derived GEMM cells are permissive to CMV infection is thus a major goal of our current investigations.

An interesting question regarding GEMM cells is: when did they arise during CD34^+^ HSC differentiation, and what factors influence this process? The CD34^+^ cells we employed in this study were isolated from cord blood and were amplified for 8–10 days in the presence of FL, SCF and TPO before differentiation. These cytokines are known to promote CD34^+^ cells self-renewal and have been used to expand HSC *in vitro* for therapeutic intervention (Piacibello et al., 1997; Gilmore et al., 2000; Tanavde et al., 2002; Flores-Guzman et al., 2013; Psatha et al., 2016), a process consistently associated with the rapid loss of the CD34 marker (Tanavde et al., 2002). In addition to stimulating division, TPO also drives megakaryocyte development (Machlus and Italiano, 2013), while FL steers hematopoiesis toward the lympho-myeloid lineage at the expense of erythrocytes/megakaryocytes, and is essential for the generation of dendritic cells (Tsapogas et al., 2017). These cytokines may thus have provided the very first “ridges,” nudging HSC differentiation toward GEMM cells. Intriguingly, we were able to detect the presence of progeny virus in the culture supernatant of amplified (but not of non-amplified) CD34^+^ cells exposed to TB40/E, albeit with low frequencies (not shown). This led us to wonder if, perhaps, GEMM cells might be present in amplified HSC cultures even before exposure to the differentiation cocktail. Moreover, the efficiency of GEMM cell generation was also shown to vary depending on the blood source, with cord blood HSC being more productive than peripheral blood or bone marrow HSC (Piacibello et al., 1997; Tanavde et al., 2002). Together, these findings clearly show that CD34^+^ cells are extremely plastic, and can easily give rise to clustered sub-populations of cells, some of which permissive to lytic infection. Being a minority in the population these clusters can easily escape detection and may introduce unwanted and unnoticed “lytic noise” (Shnayder et al., 2018) in studies of viral latency.

While the reason for the preferential infection of CMV-transcript^+^ cells remains unclear, our data provide a plausible rationale for initiation of lytic infection in GEMM cells, i.e., their higher expression of multiple gene products involved in energy, RNA, and protein production, as well as in cell cycle control. This likely create an intracellular environment particularly conducive to infection onset by lowering the amount of energy required from viral effectors to steer cellular processes away from cell needs and toward viral replication. We initially reported the up-regulation of numerous genes involved in mitochondrial energy production in infected fibroblasts at late times pi (Hertel and Mocarski, 2004). Our findings were subsequently confirmed and expanded by a number of studies in different cell types (Munger et al., 2006; Kaarbo et al., 2011; Jean Beltran et al., 2016; Karniely et al., 2016). Similarly, we (Hertel and Mocarski, 2004) and others (Tirosh et al., 2015) observed that expression of genes involved in RNA processing, splicing, and translation is induced in infected fibroblasts. Here, we show that these types of genes are also strongly up-regulated in myeloid cells and at early times post-entry (Figure 6), marking these metabolic processes as pivotal for successful CMV replication in different cell types.

The cell cycle is also a very well-known target during viral infection. We previously showed that CMV infection is associated with a strong positive impact on the expression of multiple S phase, M phase, and DNA activity regulators in fibroblasts, leading to the appearance of aberrant mitotic figures, which we called pseudomitosis, at late times pi (Hertel and Mocarski, 2004; Hertel et al., 2007). Here, we found that expression of genes involved in S phase control was higher in GEMM cells and remained high in CMV^+^ cells, whereas transcription of M phase regulators was reduced (Figure 6). While fibroblasts were infected at confluency (when the majority of the cells are in G0/G1), GEMM cells were likely actively proliferating at the moment of contact with CMV. We thus believe that our new data highlight CMV’s exquisite ability to fine tune its impact on gene transcription according to the conditions of the cell at the time of entry, in order to reach optimal expression levels of specific genes useful to viral replication. For instance, although entry into mitosis is clearly detrimental to viral replication (Eifler et al., 2014), the presence of select M phase proteins may be needed to perform specific tasks, such as viral genome compaction, disentangling, or transport. To reach an ideal protein concentration these genes may thus need to be transcriptionally upregulated in quiescent fibroblasts, whereas downregulation may prevail when cells are already actively cycling.

Finally, the direct comparison of gene expression levels in CMV^+^ and GEMM cells allowed us to detect a much stronger negative impact of infection on the expression of IFN-related genes than previously reported, accompanied by the induction of a very small, and apparently selected, set of genes. Our data thus provide a new perspective on how host defenses are raised and subsequently offset by the virus than that afforded by previous analyses comparing mean gene expression levels in CMV- and mock-infected cells (Browne et al., 2001; Abate et al., 2004; Slobedman et al., 2004; Chan et al., 2008; Mezger et al., 2009). Our data also broaden the number of IFN-related genes susceptible to transcriptional regulation by CMV to include effectors with currently no known role in CMV infection inhibition.

Aside from the detection method, the time pi, cell type, and strain of virus used may also have contributed to the observed differences. Infection recognition occurs very rapidly in monocytes and fibroblasts, leading to the activation of the transcription factors IRF3 and NF-κB, and to the implementation of the IFN transcriptional program within 4–8 h (Zhu et al., 1997; Boyle et al., 1999; Yurochko and Huang, 1999; Browne et al., 2001; Preston et al., 2001; Abate et al., 2004; Netterwald et al., 2004; Chan et al., 2008). Structural components of the virion, such as the tegument proteins pp65 and/or pp71 (Browne et al., 2001; Abate et al., 2004; Fu et al., 2017), as well as viral immediate-early and early proteins (Khan et al., 2005; Taylor and Bresnahan, 2006a) subsequently cooperate to blunt these responses (Miller et al., 1999; Taylor and Bresnahan, 2006b; Le et al., 2008; Marshall and Geballe, 2009; Verma and Benedict, 2011; Fu et al., 2017; Biolatti et al., 2018; Goodwin et al., 2018). It is thus possible for IFN-related genes to be highly transcribed at 4–8 h pi, but downregulated at 24 h pi. The importance of viral countermeasures is indeed underscored by the fact that five of the nine genes more abundantly expressed in CMV^-^ cells encode IFN-induced antiviral proteins (Supplementary Figure S2), suggesting that reduction of their expression could not be achieved in the absence of specific viral gene products.

Basal expression levels of sensors, signal transducers, and IFN-inducible genes can also vary according to the cell type (van Boxel-Dezaire et al., 2006), while the speed and strength whereby antiviral responses are blunted can be affected by the virion content of pp65, which differs substantially depending on the virus strain (Jahn et al., 1987).

Altogether, our data provide evidence in favor of the existence of a new type of myeloid cells potentially permissive to CMV lytic infection, and offer a reasonable theory regarding their preferential infection over other cell types present in the same population. These results also substantially expand our understanding of the cellular determinants of CMV tropism for myeloid cells, and provide new candidate pro- and anti-viral molecules for future studies and therapeutic interventions.

## Author Contributions

LH conceptualized the study, analyzed and visualized the data, and wrote the original draft of the manuscript. MG, KS, and AA generated and analyzed the data. FH and DB performed the Monocle analysis and visualization of the data. LH, DB, and MM reviewed and edited the manuscript, and acquired the funding.

## Conflict of Interest Statement

The authors declare that the research was conducted in the absence of any commercial or financial relationships that could be construed as a potential conflict of interest.

## Footnotes

1 https://support.10xgenomics.com/single-cell-gene-expression/software/pipelines/latest/rkit
