# Supplementary material for: Single-Cell Transcriptome Analysis of CD34+ Stem Cell-Derived Myeloid Cells Infected With Human Cytomegalovirus
Source: Front Microbiol. 2019 Mar 21;10:577. doi: 10.3389/fmicb.2019.00577 (PMC6437045; doi:10.3389/fmicb.2019.00577)
Supplement: Supplementary file 1 [file Data_Sheet_1.PDF]

Estimated Number of Cells

6,837

Mean Reads per Cell	Median Genes per Cell
44,094	2,305

Sequencing

Number of Reads	301,474,803
Valid Barcodes	97.8%
Reads Mapped Confidently to Transcriptome	69.0%
Reads Mapped Confidently to Exonic Regions	73.5%
Reads Mapped Confidently to Intronic Regions	20.1%
Reads Mapped Confidently to Intergenic Regions	2.4%
Sequencing Saturation	59.9%
Q30 Bases in Barcode	98.5%
Q30 Bases in RNA Read	95.5%
Q30 Bases in Sample Index	98.2%
Q30 Bases in UMI	98.8%

Cells

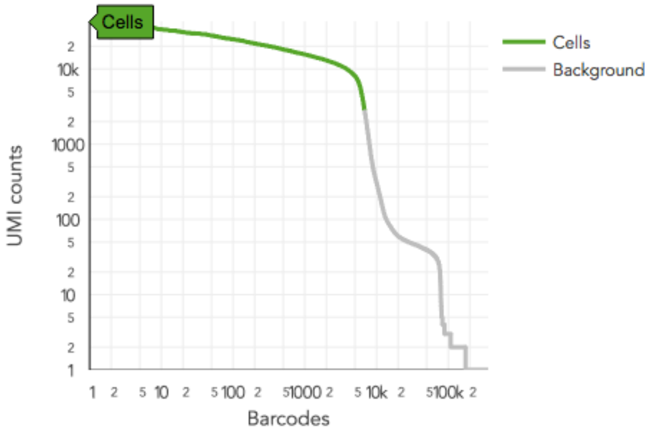

Estimated Number of Cells	6,837
Fraction Reads in Cells	92.1%
Mean Reads per Cell	44,094
Median Genes per Cell	2,305
Total Genes Detected	20,899
Median UMI Counts per Cell	10,627

Supplementary Figure 1. Summary of sequencing parameters and outcomes.
