# Supplementary material for: Single-Cell Transcriptome Analysis of CD34+ Stem Cell-Derived Myeloid Cells Infected With Human Cytomegalovirus
Source: Front Microbiol. 2019 Mar 21;10:577. doi: 10.3389/fmicb.2019.00577 (PMC6437045; doi:10.3389/fmicb.2019.00577)
Supplement: Supplementary file 2 [file Data_Sheet_2.PDF]

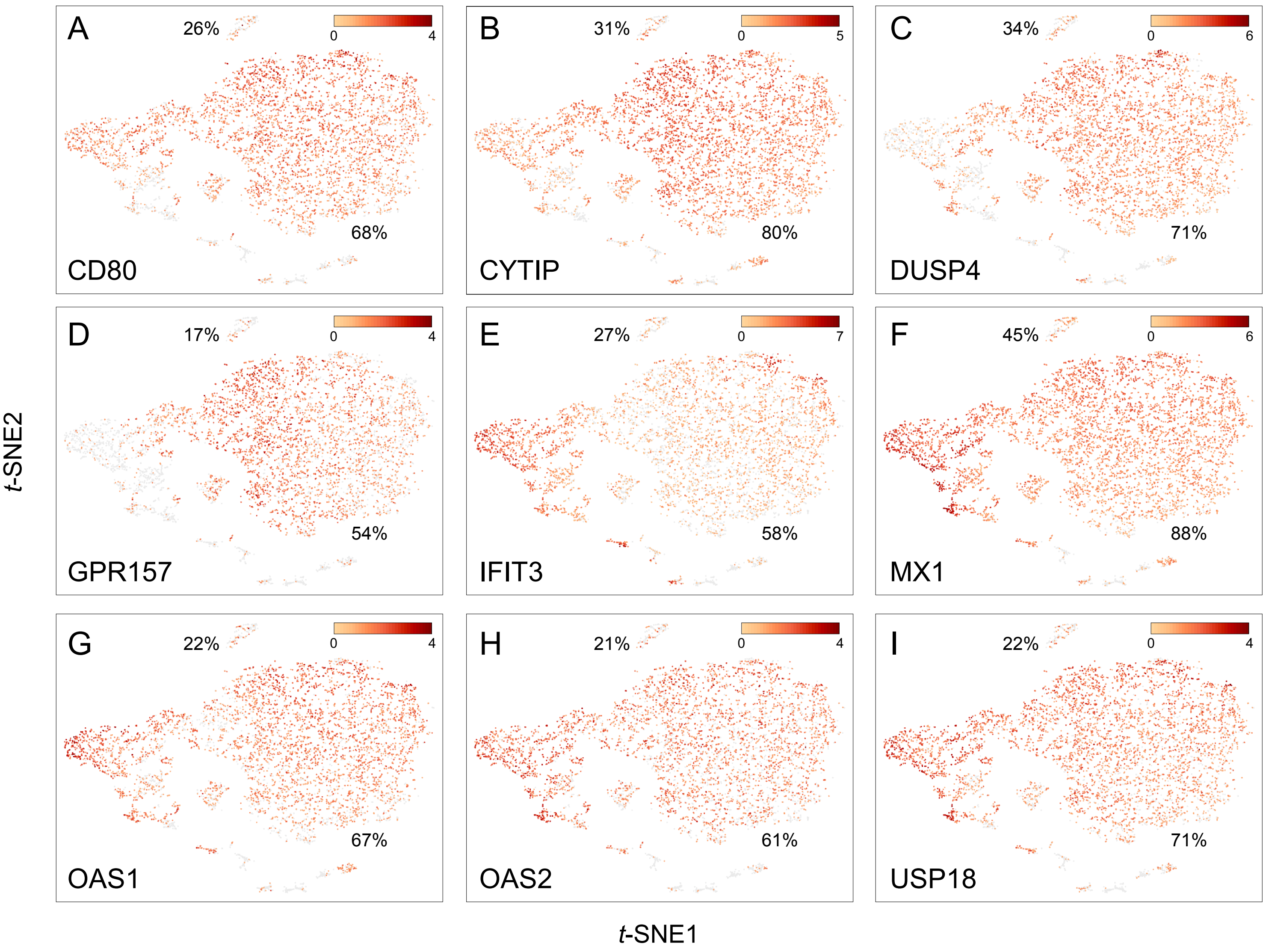

Supplementary Figure 2. Transcript abundance and distribution of the nine cellular genes with four-fold higher mean expression levels in CMV- than in CMV+ cells and present in more than 50% of CMV- cells, but less than 50% of CMV+ cells.

t-SNE projection of data from profiled cells colored based on their quantitative (Log2 Gene Exp Max) content in transcripts mapping to the cellular genes named in each box. The proportion of CMV+ and CMV- cells expressing each gene is indicated beside the CMV+ cluster and in the bottom right corner of each box, respectively.
