# Supplementary material for: Single-Cell Transcriptome Analysis of CD34+ Stem Cell-Derived Myeloid Cells Infected With Human Cytomegalovirus
Source: Front Microbiol. 2019 Mar 21;10:577. doi: 10.3389/fmicb.2019.00577 (PMC6437045; doi:10.3389/fmicb.2019.00577)
Supplement: Supplementary file 3 [file Data_Sheet_3.PDF]

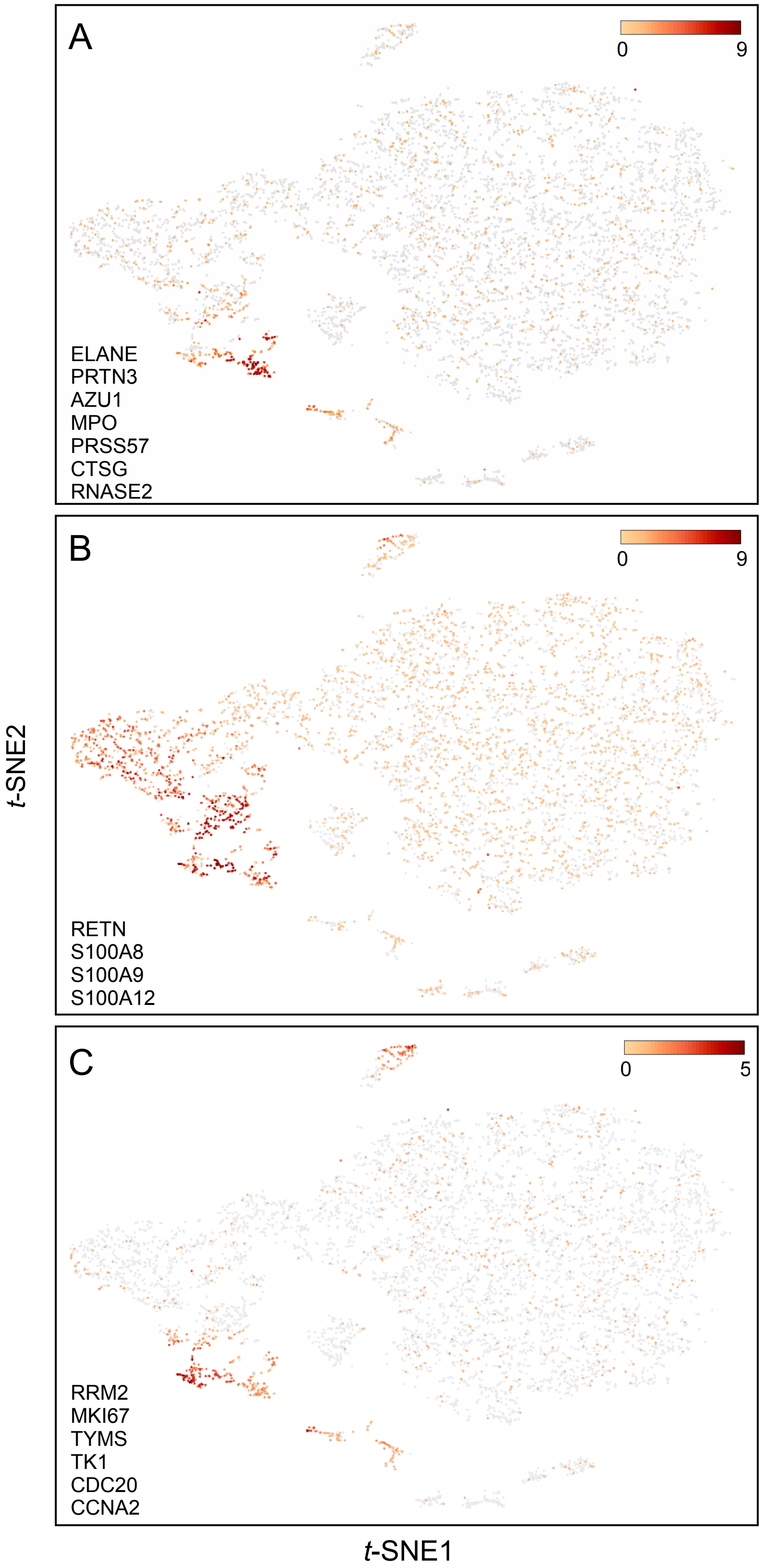

Supplementary Figure 3. Transcript abundance and distribution of cellular gene markers for promyelocytes, activated neutrophils, and sub-cluster 3 cells.

t-SNE projection of data from profiled cells colored based on their quantitative (Log2 Gene Exp Max) content in transcripts mapping to the genes listed in the lower left corner of each panel and corresponding to the promyelocytes **(A)**, activated neutrophils **(B)**, or sub-cluster 3/GEMM **(C)** clusters in Figure 3C.
