# Supplementary material for: Single-Cell Transcriptome Analysis of CD34+ Stem Cell-Derived Myeloid Cells Infected With Human Cytomegalovirus
Source: Front Microbiol. 2019 Mar 21;10:577. doi: 10.3389/fmicb.2019.00577 (PMC6437045; doi:10.3389/fmicb.2019.00577)
Supplement: Supplementary file 4 [file Data_Sheet_4.PDF]

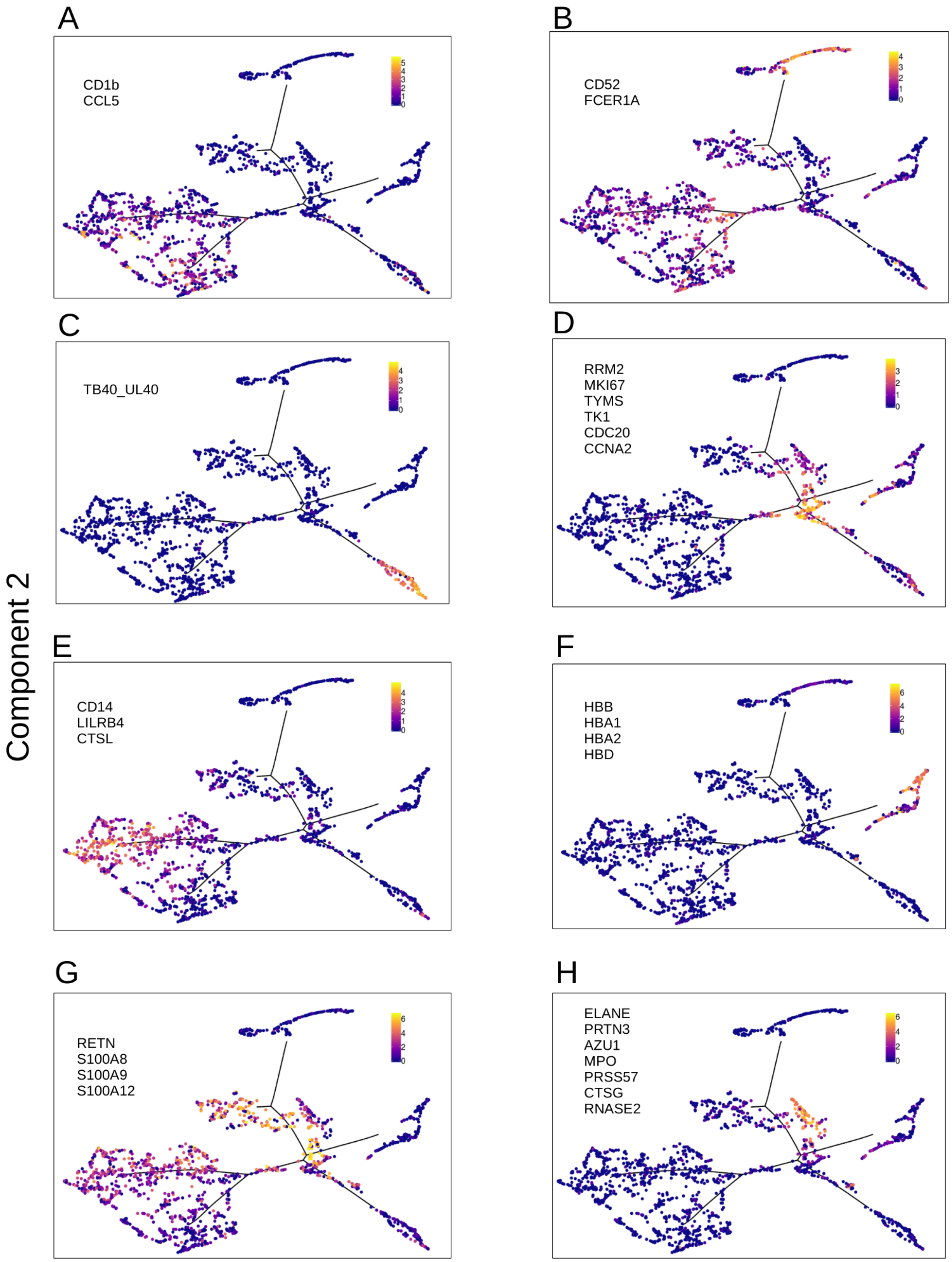

Supplementary Figure 4. Transcript abundance of gene markers used to characterize the cell groups shown in Figure 4.

Monocle pseudotime trajectory of cells colored based on their content in transcripts mapping to the genes listed in the upper left corner of each panel and corresponding to the GR A – MDDC **(A)**, GR B – CL7 **(B)**, GR C – CMV **(C)**, GR D – GEMM **(D)**, GR E – Mono **(E)**, GR F – Erythro **(F)**, GR G – Act Neut **(G)**, and GR H – Promyelo **(H)** groups in Figure 4B.
